# Disentanglement of prosodic meaning: Toward a framework for the analysis of nonverbal information in speech

**DOI:** 10.1073/pnas.2500510122

**Published:** 2025-09-12

**Authors:** Tirza Biron, Moshe Barboy, Eran Ben-Artzy, Alona Golubchik, Yanir Marmor, Assaf Marron, Smadar Szekely, Yaron Winter, David Harel

**Affiliations:** ^a^Weizmann Institute of Science, Faculty of Mathematics and Computer Science, Rehovot 7610001, Israel

**Keywords:** computational prosody, prosody, NLP

## Abstract

We propose a theoretical framework and a cost-effective automated method for the interpretation of prosodic messages (e.g., chunking of information, emphasis, conversation action, emotion). At the core of the proposal is a hierarchy of layered prosodic messages that co-occur within the same intonation unit (0.5 to 2 s long). Motivated by this hierarchy, a procedure for the differential detection of three such co-occurring nonverbal messages is then described. In way of implementation, we produce a variant model of the WHISPER automatic speech recognition system that flags intonation unit boundaries, intonation unit prototypes, and emphases therein. The procedure required us to alter WHISPER’s token combinations and significantly adjust its prediction process. The variant model was tested on four datasets that contain spontaneous and read speech, and performs on a par with similar human annotation, and often better, using relatively modest training data. Several insights regarding this implementation, such as model size and encoding methods, are described as well. We believe that the proposed framework, coupled with the results of its application herein, can greatly improve the analysis of speech and language, integrating contextual information and speaker intentions into linguistic descriptions for a large array of purposes with modest means.

## Significance

Nonverbal messages in speech - emotion, emphasis, conversation action - are encoded by prosody, providing “operating instructions” for words and contextual information. Despite its importance, the principles that govern prosodic structure remain unformulated. In this work, we present theoretical and technological infrastructures for the disentanglement of multi-layered prosodic messages. It is based on a natural unit of speech, termed “intonation unit” (IU), and its variations. Our results for a disentangled recognition of three co-occurring messages—IU boundary, IU prototype and emphasis—are 0.935, 0.72 and 0.71 (F1), respectively. In addition to enhancing context formalization, our approach can foster a standardization of prosody, directing a theory of speech organization. The results can be beneficially applied in a number of speech- and language-related technologies.

The speech signal carries not only words but also “music,” or prosody. Prosody encodes nonverbal contextual information, such as attitude and emphasis, conversation action, and emotion (see, e.g., refs. 1–3). Written text records several of the more obvious prosodic functions, such as certain kinds of segmentation (by using, e.g., commas and full stops), discourse organization cues (using dashes or semicolons), as well as emphasis (italics or boldface) and misgivings (e.g., by quotation marks). However, the vast richness of prosody leaves much to be studied (e.g. refs. 4 and 5), as do the terminology used for its description (e.g., refs. 3, 6, and 7) and the automatic detection of its messages.

Our core motivation is to contribute to context formalization in linguistic accounts, both classic and automated. We follow in the footsteps of, e.g., de Beaugrande (8) and Austin (9), who advocated studying “not the sentence but the issuing of an utterance in a speech situation” (p. 138, and see also ref. 10). This approach directs the linguist to analyze the intention and meaning of communication—feelings, objectives, and epistemological information—rather than its syntactic components. To the discerning ear, prosody reflects and imparts, remarkably and accurately, such intentions. Contextualization through prosody emerges as a natural next step.

Consider Ex. **1**:

[1] $$\begin{array}{c} \text{“There is a bull in the field.“ (9, p. 32)} \end{array}$$

This statement would ordinarily be interpreted as either a description or a warning, depending on the speaker’s motivation. Adding prosodic conditioning to the recognized words would enable speech technologies, for example, to produce outputs such as “There is a bull in the field (warning, urgent),” or “There is a bull in the field (background description, narrative, neutral),” and generate a more targeted response.

A significant difficulty in achieving this goal is the fact that different prosodic messages co-occur within the same prosodic unit. A speaker may utter a sentence that a human listener will immediately recognize i) to be a question, ii) to contain a part that is emphasized, and iii) to express unpleasant surprise—all at the same time; see Ex. **2**:

[2] “You want to go home?!”

The author of ref. 4 acknowledges this entanglement, stressing that “the heavy overlap of the communicative functions [—] makes it difficult to directly identify the unique contribution of each function to surface prosody” (see also the introduction in ref. 2). Toward addressing this question, we present a procedure for the interpretation and disentanglement of prosodic messages.

First, we propose a theoretical framework based on a layered structure of prosodic functions/messages, in which each layer represents a different prosodic category. These categories are designed to be general and to capture fundamental semantic dimensions of prosody that are consistent across datasets. Within each category, there are individual labels/ subcategories that may be dataset-dependent, reflecting specific linguistic or cultural variations [e.g., upspeak (11) as conversation action]. This approach allows for both a standardized analysis across speech communities and the flexibility to adapt to community-specific prosodic systems.

As basic units for analysis, we propose the short chunks of speech termed intonation units (henceforth IUs). IUs have been described either by a set of prosodic cues, or by their function of parsing speech and delivering one principal item of information (12–15). They are also known to be correlated with brain activity (16). Essentially, our procedure provides classification trees for IUs.

Based on this theoretical framework, and to show that co-occurring prosodic messages may be detected simultaneously, as they are formed in speech, we describe a fine-tuning process and produce a variant model of WHISPER (17) for the differential analysis of nonverbal messages. The model was retrained to detect three prosodic functions: IU boundaries, IU prototypes (see below), and emphases, by applying a new set of token combinations.

As will be shown, this transfer learning required us not only to alter WHISPER’s token combinations but also to ignore the original decoded ones. We believe that the model is thus provided with better conditions for mapping prosodic phenomena. To the best of our knowledge, these means have not been employed for multitask/multilabel prosody recognition and prosodic disentanglement.

Although our aim was not to achieve exceptional performance, our system, when tested on spontaneous and read American-English speech, performed on a par with or better than human annotation on similar tasks (cf. ref. 18).

Since the disentanglement of IU patterns is a new problem, we found it necessary to present two American English datasets that have been meticulously annotated with the three above-mentioned prosodic functions. The annotations reflect our focus on prosodic messages/functions rather than prosodic form.

In addition to an explication of IU pattern and function, the disentanglement of prosodic messages can shed light on the organization of speech; it may ultimately enhance the pairing of prosodic form and function (cf. ref. 7); i.e., determine which part of the speech signal (form) is associated with which prosodic meaning (function). Furthermore, since prosody reflects a large part of the communicational context in speech, its automated analysis can significantly improve context formalization for speech-related technologies. Such improvements include better deciphering of speaker intentions for bots, automatic translation, meeting summarization, aids for the hard of hearing, etc.

## Related Work

### Linguistic Approaches.

For overviews of the prevalent linguistic approaches to the study of prosody, see, e.g., refs. 2, 5, 7, 19, and 20. An influential description of prosodic form is the Autosegmental/metrical approach (henceforth ASM) and its corresponding annotation scheme, ToBI (tones and break indices) (21). It presents a hierarchy of prosodic elements, from the mora to the intonational phrase (3); however, it does not account for the multiple, co-occurring prosodic messages in spontaneous speech, nor does it consider prosodic communicational function and meaning, which are the object of our study and the focus of our approach.

As for the study of prosodic functions and meaning, whether text-combined or not, it is common to look at linguistic and paralinguistic categories, such as unit boundary, emphasis, discourse function, and emotion, as mentioned above (see also refs. 22 and 23). In ref. 24, for example, the authors stress prosody’s central role in conversation analysis, and describe several distinctly marked patterns, as do (25, 26).

More comprehensive views on prosodic meaning/semantics are presented in refs. 1 and 22. CALLIOPE (22) offers a conceptual model for the analysis of prosody, that holds twelve dimensions, each defined by its independent contribution to the acoustic realization of an utterance (e.g., type of illocutionary act, dialect, pragmatic focus, or rhetorical form). The PENTA model (1) proposes an interpretation of prosodic meaning as layered signals (see also ref. 27). We concur with both approaches, but propose a different analytical structure and prosodic layering, specifically in relation to the hierarchy of prosody production and the number of dimensions to be interpreted (*Theoretical Linguistic Framework*).

Like Xu et al. (1), we adopt the functional approach to prosodic units: it is function rather than form that is the primary guide to the delineation of prosodic units (1). In our case, this extends to IUs as functional entities (13). Note, however, that the definition in ref. 13 may not neatly conform to the ASM approach (21). Thus, a point of difference with refs. 1 and 22 regards the unit to be analyzed—a crucial decision in prosodic research: PENTA (1) deals with pitch targets as elementary components for encoding prosodic functions, whereas CALLIOPE (22) uses “information units” (which roughly correspond to sentences). Our framework, however, stipulates a different scope of patterning.

### Automatic Prosody Recognition.

In close correlation with most linguistic approaches, automated prosody recognition does not undertake the detection of multiple, co-occurring prosodic messages. An exception to this rule is described in ref. 29, and see below. Algorithms, whether text-assisted or not, usually assign single labels from the following categories:

- **Prosodic boundary**: Existing algorithms detect tone group, prosodic phrase, intonational phrase, or intonation unit (IU), e.g., refs. 30 and 31. A direct predecessor of our method is PSST (32), in which the authors enrich an automatic transcription with boundary labels, retrained on the Santa Barbara Corpus (SBC) (28). Apart from the difference in task (single vs. triple recognition), the PSST method is designed to predict IU boundary conjointly with transcription. Our method avoids the latter, overriding this component, as we estimate that the model’s ability to map prosodic phenomena is thus improved.
- **Dialogue act and sentence modality/parasyntax**: e.g., ref. 33 uses prosodic features to classify declaratives, interrogatives, and exclamatory sentences; PESInet (34) predicts the punctuation at the end of sentences based on text and audio.
- **Emphasis/saliency/stress/prominence**: Of the many methods for the automatic detection of prominence/saliency, we cite but a few: ref. 35 for pitch accent detection; ref. 36 for the relations between f0/pitch and (syllable-sized) duration contours; ref. 37 for sentence-prominence detection; and refs. 38–40 describe the detection of prosodic prominence using either prosodic/ acoustic features and/or a combination of those and part-of-speech information.
- **Syntactic/pragmatic entity recognition**: Pragmatic components such as topics were identified in, e.g., refs. 41 and 42, offshooting the seminal work by Shriberg et al. on topic recognition (43).
- **Emotion**: The recognition of emotion and sentiment through speech and prosody has attracted much attention over the past decades (see refs. 44 and 45). Many researchers (e.g., refs. 46–48) represent emotions as coordinates in a three-dimensional space of arousal, valence and dominance. Others use the 7-emotion scheme by Ekman (49). Training is most often carried out on acted speech. The dataset described in refs. 48 and 50, however, consists of spontaneous emotion. In recent work (51), the authors researched the benchmark for self-supervised learning (SSL) on prosody-related tasks, which include the detection of sarcasm and persuasiveness.
- **Multitask recognition**: The above-mentioned efforts detect prosodic events as single recognition tasks. Detection of more than one event, such as IU boundary and stress together, has been done, e.g., in refs. 29 and 52 on British English broadcast news. The latter method incorporates, in addition to ToBI annotation, part-of-speech labels, and uses B-directional long/short-term models (BLSTM) on aggregated features for phones, syllables, and words. It is a method that is specifically tailored to the Aix-MARSEC (53) and similar ToBI datasets, and requires quite extensive preparation. Conversely, our method of per-word prosodic label predictor (henceforth WPLP) is easy to implement, with robust performance on concurrent tasks.

### Machine Learning and Disentanglement of Meaning.

Disentanglement of signals is tackled by ML methods in a variety of domains. In computer vision and image processing, for example, it is used for distinguishing content from style, as well as visual concept tokenization (e.g., refs. 54–56).

Speech-signal decomposition is performed regularly for automatic speech recognition (ASR), for example, by excluding parts of the signal that are not associated with word production (57). In the domains of speech generation and synthesis, disentanglement is aimed at discovering speech representations by modeling text and prosody, separately or together (e.g., refs. 58 and 59).

As stated above, to the best of our knowledge, the multiclass/multilabel transfer learning that we employ has not been used for prosody analysis.

## Theoretical Linguistic Framework

**Fig. 1.**
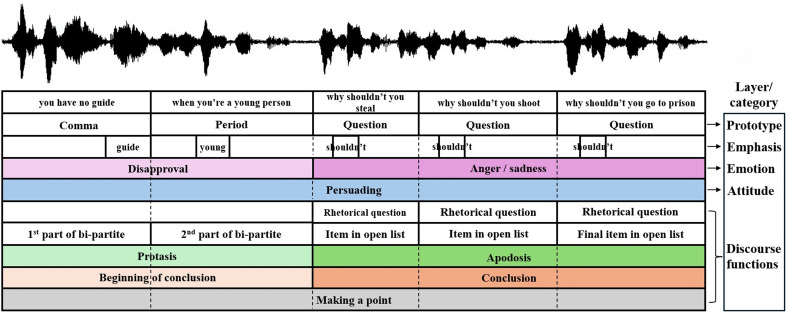
An example of contextual information as represented by categories and labels of prosodic messages in an excerpt from the Santa Barbara Corpus of American English (28), henceforth SBC. Our annotation is function-based rather than form-based (FUNCrFORM).

**Fig. 2.**
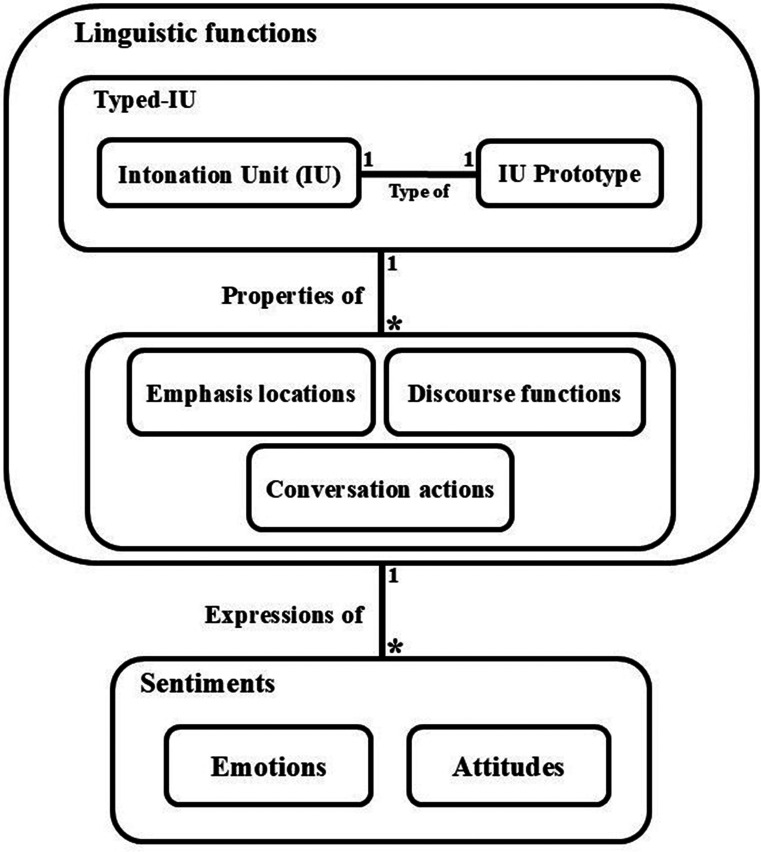
A hierarchical diagram of the various kinds of prosodic elements/messages that are dealt with in the paper. An IU is a 0.5 to 2.0 s spurt of speech—a widely accepted basic element for prosodic analysis. The IU Prototype indicates the type of each IU: continuation, conclusion, or request for response, denoted respectively by a comma, a period, or a question mark (the connecting edge between them is adorned with 1’s, indicating the 1–1 nature of the relationship). The two taken as a whole, constitute what we shall call a Typed-IU (TIU). The Emphasis locations denote a property of the TIU, indicating which part or parts of it is/are emphasized by the speaker; i.e., is/are marked as salient. Discourse functions, also properties of the TIU, include various possibilities of the rhetorical and discourse-structural characteristic thereof, such as a parenthetical phrase, part of an if-then structure or a list of items. Conversation actions, such as a command, a request, or a rhetorical question, are also a linguistic property of the TIU. A TIU may be associated with several discourse functions (this potential multiplicity is depicted in the diagram by the *, which denotes zero or more). Emotions and Attitudes are the subtler semantic prosodic elements, and express sentiments associated with the TIU in question. Emotions include anger, sadness, surprise, joy, etc., and Attitudes include feigned surprise, sarcasm, attentiveness, etc.

In this section, we present our framework for a layered prosody analysis (see example in Fig. 1, and to help follow this section, see Fig. 2). The categories suggested below are seen as applicable across datasets and speech communities, whereas subcategories/individual labels (as in the main part of Fig. 1, for example) are considered to be dataset specific. At its heart is a hierarchy of IU patterning.

Three premises direct the proposal:

(i) that an IU is a suitable unit for identifying intelligible, cohesive prosodic messages. It is a functional speech entity on a timescale that is similar to a phrase, and thus more readily interpretable than its parts (e.g., syllables);
(ii) that IUs convey communicative functions—semantically meaningful, sometimes grammaticalized, prosodic patterns (24, 60, e.g., Chapter 4);
(iii) that “prosodic means [-] are often encoded by modifying existing forms that are already specified by other functions” (1).

The “existing forms” in this case are 3 to 4 fundamental IU prototypes that exhibit, in turn, meaningful prosodic variations. By the term “prototype” we mean an underlying prosodic pattern or template, which is an attribute of every IU and a prerequisite for every additional prosodic information. Maintaining enough of its essential features, it serves as a listener’s guide to infer the rest of the speaker’s nonverbal intentions (cf., ref. 28 for IU types that are form-rather than function-based, henceforth FORMrFUNC). Maintaining enough of its original features, it serves as a listener’s guide to infer the rest of the speaker’s nonverbal intentions. Consider example no. **2** once again:

[2] “You want to go home?!”

Here, the prototype request for response [?] (see below) is the underlying, existing form that remains identifiable through the speaker’s other messages (discontent; assertive; asking a rhetorical question to challenge; and emphasizing the word “home”). Such messages are grafted, so to speak, onto the prototype template. For this basic entity, of an IU that is paired with its prototype, we propose the term “Typed-IU” (henceforth TIU), and suggest three basic TIUs: “continuation,” “conclusion,” or “request for response,” denoted respectively by a comma, a period, or a question mark.

According to our framework, for the data that we have examined, each TIU can convey 3 to 8 (but no more) layers of variation on 3 to 4 basic TIU prototyped-templates. By “layers of variation” we mean TIU pattern-variation of several orders of information: 1) discourse function and/or conversation action; 2) emphasis/information structure; 3) express attitude; and 4) unintended/unplanned emotion. The first two categories are linguistic, whereas the latter two are sentiment-related. Fig. 2 describes the relationships between these prosodic messages/dimensions (22).

We consider the prosodic IU prototype to be an equivalent of parasyntax/modality, akin to the difference between a statement and a question [cf. refs. 13 and 14 for a FORMrFUNC annotation scheme, and see also (23, 61, 62)].

**Fig. 3.**
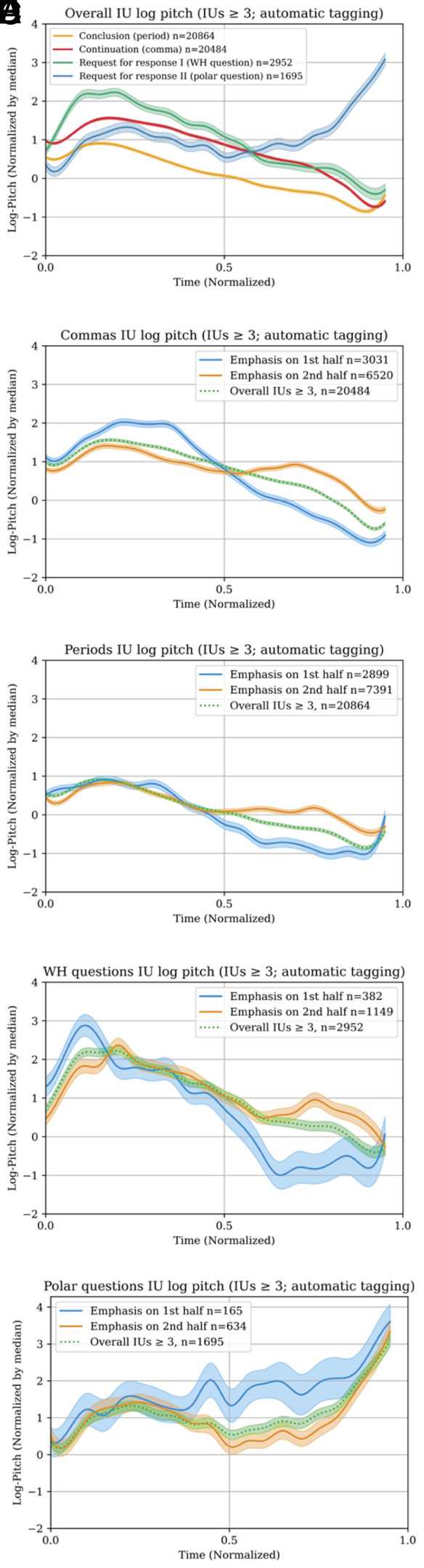
Underlying IU prototypical patterns/TIU templates and the prosodic analytical hierarchy: plots present the normalized median F0/pitch values in semitones for each time-normalized TAL dataset TIU of 4 to 7 word length, automatically annotated by our WPLP model. The median F0/pitch is represented by the dark shade, whereas the lighter shade represents the error bars. (*A*) The normalized pitch courses of the unmarked prototypes. (*B*–*E*) The production of emphasis in the beginning vs. in the end of three IU prototypical patterns: (*B*) Continuation [,] (n = 20,484); (*C*) Conclusion [.] (n = 20,864), and request for response [?] divided into (*D*) WH-questions (n = 2,952) and (*E*) Polar-questions (n = 1,695). Note that the pitch maxima in the beginning of the IU vs. its end echo the underlying pitch template. For a detailed description of the production of Fig. 3, see *SI Appendix*, section 2.

Fig. 3 demonstrates the effect of the prototype template. It shows the statistical interaction between underlying pitch contours and the production of emphasis in two positions: in the beginning vs. the end of an IU. Fig. 3*B*–*E* present the emphases against the backdrop of the unmarked prototypes of “continuation [,]”, “conclusion [.]”, “request for response I/WHquestion” [?], and “request for response II/Polar-question” [?], respectively. The emphasis pitch maxima reflect the prototype template, confirming the hierarchical relationship between these two prosodic dimensions (cf. ref. 27).

The formalization of IU patterning can thus advance toward disentanglement and differential flagging. When the underlying pattern of an IU is identified, other prosodic messages may be identified as well: a question pattern can be extricated from the pattern of disapproval and that of emphasis, etc.; this, in addition to other phonological and prosodic constraints, such as syllable structure or unit length (see, e.g., ref. 63), that together influence prosody production.

The following is a brief explanation of the four prosodic categories of variation that can be manifested within the TIU (and see *SI Appendix* for a more detailed description).

### Discourse Function and Conversation Action.

We consider this category to be closely related to the prototype one. Clearly, the prototypes continuation [,], conclusion [.] and request for response [?] play a significant role in discourse organization; however, the category of “discourse function” adds information in regard to the relationship of one TIU with other TIUs when forming larger structures. For example, in “you snooze, you lose”, the if-clause will usually employ the continuation [,] prototype, whereas the then-clause will use the conclusion [.] one.

The analysis of discourse covers a wide range, from syntax to rhetoric (see proposed schemes in, e.g., refs. 22 and 64–66). The interpretation that we offer for prosodic discourse organization is, as of yet, incomplete: there are more categories and properties of TIUs, as well as interrelations between subcategories, that need refining. TIU tags include, for example, “circumstantial unit,” “title of discourse,” “background to narrative,” and “narrative event.” Some examples, cited from our datasets, can be found in *SI Appendix*, Table S4 and Fig. S1.

Within this category, we add the subcategory of “conversation action,” which refers to speech acts that are directed at influencing the interlocutor’s behavior (warnings, commands, apologies, complaints, questions that serve as requests, etc.). Indeed, for good measure, it would be best if one could neatly distinguish conversation actions from discourse functions. However, this is often not the case. For additional details, see *SI Appendix*.

### Emphasis and Information Structure.

The prosodic signaling of saliency of information (un-/de-/secondary-/emphasis, e.g., ref. 67): emphases are the signal variations that produce differences, e.g., between new information (“I’m a doctor”), or contrastive emphasis (“I’m a doctor”).

### Express Attitude.

Irony, reserve, feigned anger, and calculated indifference are examples of overt attitudes, as are mock surprise, empathy, sarcasm, etc. (e.g., refs. 68 and 69).

### Unintentional/Unplanned Emotion.

This category includes, for example, delight, disgust, fear, pain, anger, sadness, or disappointment, as well as other emotions that can alter one’s prosody (see overview in refs. 44 and 70).

### Concluding Remarks.

We draw our labels from the vast body of linguistic knowledge in pragmatics, text-linguistics, conversation and discourse analysis, phonology, as well as psychology; e.g., refs. 24 and 71–74. Together with reviewing large amounts of speech data, the categories and subcategories that these domains offer led us to establish our own (see example in *SI Appendix*, Tables S3–S5). Yet, we acknowledge that our set of labels—subcategories—is incomplete.

Some prosodic messages are rather subtly marked, while others are more clear-cut (e.g., discourse function vs. emphasis/information structure). When analyzing speech, our description of these messages strives to be as detailed as possible.

From a strictly theoretical perspective, our framework is a structuralist-contrastive tool: it is a description of a semiotic system in the classical way (75). A prosodic category is thus defined by whether or not it can co-occur with other categories in the same TIU (e.g., emphasis and emotion). Subcategories or individual labels, on the other hand, are mutually exclusive members of one category (e.g., prototypes: three members; information structure category: four members—contrastive, informative, secondary, de-emphasis; etc.). In this sense, the theory is general and accommodates the multilayered nature of the data. Subcategories, however, need adjusting to specific datasets and, on occasion, to the purposes of classification.

From a more narrow structural perspective, our view on prosodic template-variation is akin to nonconcatenative morphology: word formation that is analyzed as the fusion of several morphemes into an amalgam signal (see ref. 76, section 3.1.1ff). When applied to IU patterning, it enables a substantial reduction in complexity—from a seemingly infinite prosodic variation to a hierarchical system.

Prosodic message-boundaries can be the beginning and ending of IUs (13; see also refs. 12 and 76–80, and cf. ref. 81 on prosodic caesurae). Although we focus in this paper on the IU, switches between- and overlaps of prosodic messages may occur on various scopes, from the syllable to larger units. For example, the messages “surprised question” may switch next to a “discontent statement.” Conversely, the switch between an emphasized word to an un-emphasized one occurs under the overlapping message of “surprised” + “question”.

In addition, and generally speaking, the layered structure that we describe leads us to forgo, for now, an attempt to attribute specific parts of the speech-signal to a distinct prosodic message. In this work, we consider the disentanglement that occurs within the model to be a satisfactory result.

The following sections report upon the methods for, and results of, a successful disentanglement procedure for three prosodic dimensions/messages—IU boundaries, IU prototype, and emphasis—as detected using the fine-tuned variant of WHISPER.

## Materials and Methods

### Datasets.

Automatic classification of multidimensional prosodic messages has attracted little attention in the ML community (see ref. 54). Since existing datasets and benchmarks do not match our analytical framework and subsequent annotation, we test our method on two specially designated datasets, tagged in-house by two expert annotators.

The principal set was drawn from the “This American Life” podcast [abbreviated TAL; (82)^*^], which contains 2 h and 10 min of read and spontaneous speech (∼60% and ∼40%, respectively). An auxiliary subset of TAL questions was prepared in order to produce Fig. 3*A*, *D*, and *E* (see *SI Appendix*, section 2 for a detailed description).

A second set was compiled for validation purposes only, consisting of 30 turns of spontaneous speech from recorded interviews. The Interviews dataset for Prosody Analysis (100) (henceforth Interviews) contains audio from seven speakers, totaling 11.5min, each audio file divided into 1 to 7 turns per speaker. Transcripts were generated by two annotators, preserving disfluencies and stutters. The annotation for validation purposes was done by a different expert than that of the TAL dataset.

We used a third set, Aix-MARSEC (53), consisting of 5 h and 32 min of mostly British English read speech (broadcast news and presentations) in order to compare our performance with that of ref. 29. In addition, for this set, we detect IU boundary and use the annotation of boundary strength as prototype-proxies to our prototypes continuation [,] and conclusion [.] (see also *Manual Annotation*, and Table 1 for prototype data). As for emphasis annotation, since the ToBI scheme is FORMrFUNC inclined [e.g., five types of pitch accents (83)], it was not used.

**Table 1.** The annotated data

(*a*) *Main speaker vs. interviewee*			
	IU count (Fraction)		
Speaker	Aix-MARSEC	TAL	Interviews
Interviewee	1,529 (12.64%)	4,551 (76.67%)	286 (66.67%)
Main speaker	10,566 (87.36%)	1,385 (23.33%)	143 (33.33%)
Total	12,095	5,936	429
(*b*) *Prosodic prototypes*			
	IU count (Fraction)		
Prototype	Aix-MARSEC	TAL	Interviews
Continuation (comma)	8,785 (72.63%)	3,246 (54.99%)	344 (80.19%)
Conclusion (period)	3,100 (25.63%)	2,362 (39.79%)	68 (15.85%)
Request for response (question mark)	210 (1.74%)	310 (5.22%)	17 (3.96%)
Total	12,095	5,936	429
(*c*) *Emphasis tags*			
	IU count (Fraction)		
Emphasis	Aix-MARSEC	TAL	Interviews
Primary	–	5,320 (26.34%)	391 (20.50%)
Secondary	–	2,726 (12.99%)	–
Nonemphasized words	–	12,946 (61.67%)	1,516 (79.50%)
Total	–	20,992	1,907

(a) IU count and fraction of main speaker data vs. interviewee data; (b) IU count and fraction of prosodic prototypes; (c) IU count and fraction of emphasis tags, i.e., the number of words annotated.

The fourth dataset, The Santa Barbara Corpus of Spoken American English (SBC) (28) consists of approximately 20 h of spontaneous speech. The annotations that we used include IU boundary and IU boundary type. The latter were considered as equivalents of our prototype annotation.

All four datasets include manual transcripts that were partially timestamped.

### Manual Annotation.

As mentioned above, our tagging of the TAL and Interviews datasets is based on the function of prosody in the instances to be annotated (IU boundary, IU prototype, emphasized words) rather than on the acoustic form that they take on in the speech signal. The annotation guidelines were quite simple: i) find IU boundary; ii) when the IU is defined, assign one of three prototypes (continuation [,], conclusion [.], or request for response[?]); iii) find the primary emphasis, if there is one; iv) if you find a secondary emphasis, mark it as well; v) you may mark the strength of the emphasis, if you find it necessary.

Interannotator agreement for the three tasks was measured on the Interviews dataset with a Cohen’s Kappa of 0.866 for IU boundary annotation, 0.728 for IU prototype, and 0.833 for emphasis. More details can be found in *SI Appendix*, section 3.

The procedure that prepares the TAL data for annotation begins with an automatic segmentation of the transcripts into word sequences that are bounded by punctuation marks. These were regarded as TIU-proxies; i.e., an entity that may possibly be a TIU. In a preliminary investigation, about 80% of the sequences that contain ≤7 words were found to correspond to TIUs, representing ordinary interannotator agreement between the TAL transcribers and our tagging (cf., e.g., ref. 84). Due to poor agreement, sequences that were eight words or longer were excluded from the TAL data. Sequences of ≤7 were thus presented as a suggestion, to be confirmed or corrected by the annotator.

The annotation of Aix-MARSEC is described in ref. 53 and, generally speaking, it uses the ASM/ToBI scheme—accent tones and break indices (21). As some basic prosodic functions can be more directly linked with form than others (e.g., ToBI breaks 3 and 4), we consider these boundary and boundary-strength tags to be useful equivalents of IU boundary and two of the three IU prototypes, continuation [,] and conclusion [.]. The third prototype, request for response [?], is rare in this set (~2%) and was therefore not considered. As mentioned above, we did not use the tonal stress markup (83), since it does not directly correlate with emphases as we tag them.

### Preprocessing for Per-Word Automatic Labeling and Training.

For normalizing TAL transcripts, that is, for obtaining a transcript of lower-case words, free of abbreviations and digits, we used the algorithm provided in ref. 85. For our purposes, the sign [—] in the original transcript was replaced by [,], and [!] by [.]. Background music was removed using the method in ref. (86). TAL and Interviews processed transcripts were then time-aligned using the Montreal Forced Aligner (87), the Aix-MARSEC transcripts using Kaldi (88), and the SBC (28) transcripts using the method described in ref. 89.

The resulting TAL and Interviews data include time-aligned words with the following tags: i) IU boundary/nonboundary; ii) IU prototype: one of three tags: [,],[.],[?] (representing continuation, conclusion and request for response, respectively); iii) saliency: emphasis and nonemphasis (see Table 1 for annotated data information). Since Aix-MARSEC tags include only boundary and two boundary types, models that were trained on Aix-MARSEC detect IU switches and prototype-proxies, and not emphasis tags. Similarly, for the SBC, we used IU boundary annotation and the form-dependent annotations for boundary tone as prototype-proxies.

### Speech-Chunk Compilation for Training and Testing.

The speech chunks that we produce are meant to somewhat emulate naturally occurring speaking turns. In speech recognition scenarios, the manner in which turns are obtained (e.g., using silent pauses, switches between speakers, etc.) implies that they begin with an IU boundary. Although not guaranteed, this is a solid assumption. Thus, our compilation induces chunks in which the first word is the beginning of an IU. The last word, however, may or may not be the end of the IU.

The considerations for obtaining suitable speech chunks were as follows (and see a pseudocode in *SI appendix*, Fig. S4):

i) Chunks should contain at least two IUs by the same speaker, so that the model may learn IU switches; ii) chunks should not contain long pauses, for efficiency of computation and to avoid IU switches that are too obvious; iii) the presence of multiple speakers in a chunk may be beneficial, as it better reflects real-life speech situations; and iv) chunks should not exceed 30 s or 448 tokens, as per WHISPER’s requirements.

In the resulting TAL data, 80% of the speech chunks are shorter than 10s; 88% are uttered by one speaker, 11.5% by two, and 0.5% include three speakers. In the Aix-MARSEC dataset, however, the vast majority (over 90%) of the recordings feature a single speaker, and 85% of the speech chunks are longer than 27s. The significantly shorter length of TAL chunks, compared to those of Aix-MARSEC, stems from the eliminations of IU-proxies that are longer than seven words (*Manual Annotation*).This created “holes” in the continuum of speech, which left the training material with chunks that were immaturely cut.

### Test Objectives and Implementations.

The primary objective of our experimental test-runs was to assess whether and to what extent a model may learn several, co-occurring, types of prosodic messages. To this end, we applied transfer learning to fine-tune an ASR model.

### Training vs. Validation Sets.

The splits into training and validation sets were done as follows: TAL was split randomly into training and validation by 85% vs. 15%, respectively. In this case, we used a larger training set in view of the modest size of this dataset. Aix-MARSEC split rates were 70%–30%, matching the setup in ref. 29. For the SBC, we assigned the first two audio files (∼40 min.) for validation, while the rest were used for training. Once again, this was done in order to match the setup in ref. 32. The Interviews dataset was used for validation only and served zero-shot testing.

### Testing Setup.

The transformer that we chose as the backbone model for our tests was WHISPER (17), an ASR tool that is based on the transformer architecture (90). To adjust WHISPER to our needs, some alterations were applied, see the subsections of Encodings of Prosodic Labels, Training and Prediction/inference.

- **WHISPER model sizes/architectures:** WHISPER available versions span from Tiny (a 39M parameter model) to Large-V2 (1.5B parameters). All versions are trained in an identical manner, except for some modification of the learning rates, i.e., smaller rates for larger models. For training the Large-V2 version, the authors of ref. 17 used more epochs and additional regularization.
- **Triple vs. single tasks:** To better understand the capabilities of the model, we compared its performance on the triple tasks (IU boundary, IU prototype, and emphasis) with that on the single tasks.
- **Encodings of prosodic labels:** To guarantee the consistency of our method, we had to verify a 1:1 mapping between the model’s tokens and the prosodic (acoustic) events. We tested two types of prosodic tag representation:
  ‒ *Compact encoding*: a specific letter is assigned to each combination of prosodic tags, represented below by *t*. Since there are 12 prosodic combinations (2 IU boundaries × 3 Prototypes × 2 Emphasis statuses), they are simply represented by the letters A to L. For example, *t* >> A:=no IU switch, question segment, no emphasis; *t* >> B:=no IU switch, comma segment, no emphasis; etc.
  ‒ *Bits*: each prosodic phenomenon is assigned a “bit,” and the combination of bits is assigned to each word, for example, *t* >> [010:=no IU switch, question segment, no emphasis]; *t* >> [020:=no IU switch, comma segment, no emphasis]; etc. Results for the Bits encoding can be found in *SI Appendix*, Table S7.

### Training.

We used the HuggingFace WHISPER implementation; the default optimization procedure is described in ref. 91. The training is done similarly to other domain- or language specific fine-tuning, by feeding the model with two-tuples of the form (signal, ground truth transcript). We therefore prepared our data such that in the ground truth transcript, each word is preceded by its corresponding prosodic tag *t*. The tag *t* represents encoded prosodic information as described above, in the Encodings of prosodic tags section, thus:

[3] $$\begin{array}{c} \begin{array}{cc} \mathrm{good morning}\;\mathrm{more news} & \Rightarrow \\ & t1\;\mathrm{good t}2\;\mathrm{morning t}3\;\mathrm{more t}4\;\mathrm{news} \end{array} \end{array}$$

This was done in order to encourage the model to detect the prosodic tag before it recognizes the word (*SI Appendix*, Figs. S1 and S2).

Each chunk of speech was treated as a single instance. Training for single tasks of boundary, prototype, or emphasis recognition required only a minor change in the above-described scheme.

### Prediction/Inference.

During inference, we generate only prosodic tokens, requiring that the model execute this task alone. Thus, in practice, we do not combine speech recognition and prosody detection (cf. ref. 32). The model predicts the sequence of prosodic tags *t*, given a signal and a transcript:

[4] $$\begin{array}{c} \mathrm{system}(\mathrm{signal},\;\mathrm{transcript})\Rightarrow t1\;t2\;t3... \end{array}$$

We thus override the decoder component (90). The decoder predicts iteratively, for each word in the already-known transcript, its corresponding prosodic label-combination. It then concatenates the tokens associated with the predicted label and the word to the accumulating series of tokens, which serve as the “footing” for the next prediction and so on (*SI Appendix*, Fig. S5). The output of the model is the sequence of the predicted labels.

### Evaluation.

We use F1 scores as the primary evaluation metric (*SI Appendix*, Table S7). Prototype performance was calculated only for the correctly identified IU (∼94% of the units); the score was based on the predicted label for the first and last words of the IU, since we found no difference in performance between representing a prototype by its first word, its last word, or both (cf. ref. 92).

## Results

### Single Dataset Performance.

#### The TAL-trained models.

The WHISPER prosodic variant models for the simultaneous detection of three prosodic messages per-word proved very successful, with encouraging performance. Predicting IU boundaries was the easiest of the three tasks, whereas detecting prosodic prototypes and emphases proved more demanding (Table 2, rows 3a to 5a).

**Table 2.** Results and comparisons—F1 scores for various detection tasks, models, and datasets

(*a*) *WPLP model size impact*								
No.	Model size	Validation Set	Task	IU	Emphasis	Comma	Period	Question
1a	Tiny	TAL	IU,Emphasis,Prototype	0.687	0.622	0.599	0.543	0.167
2a	Base	TAL	IU,Emphasis,Prototype	0.825	0.610	0.502	0.604	0.426
3a	Small	TAL	IU,Emphasis,Prototype	0.922	0.649	0.767	0.684	0.522
4a	Medium	TAL	IU,Emphasis,Prototype	0.935	0.667	0.755	0.630	0.378
5a	Large	TAL	IU,Emphasis,Prototype	0.935	0.710	0.769	0.720	0.688
(*b*) *WPLP performance for triple vs. single task*								
No.	Training Set	Validation Set	Task	IU	Emphasis	Comma	Period	Question
1b	TAL	TAL	IU,Emphasis,Prototype	0.935	0.710	0.769	0.720	0.688
2b	TAL	TAL	IU	0.946	–	–	–	–
3b	TAL	TAL	Emphasis	–	0.700	–	–	–
4b	TAL	TAL	Prototype	–	–	0.789	0.740	0.571
(*c*) *WPLP performance of models trained on other datasets*								
No.	Training Set	Validation Set	Task	IU	Emphasis	Comma	Period	Question
1c	TAL	TAL	IU,Emphasis,Prototype	0.935	0.710	0.769	0.720	0.688
2c	Aix-MARSEC (53)^†^	Aix-MARSEC	IU,Prototype	0.886	–	0.930	0.799	–
3c	SBC (28)	SBC	IU,Prototype	0.853	–	0.800	0.633	0.558
(*d*) *Comparison vs. baselines*								
No.	Method	Validation Set	Task	IU	Emphasis	Comma	Period	Question
1d	WPLP	TAL	IU	0.946	–	–	–	–
2d	WPLP	Aix-MARSEC	IU	0.895	–	–	–	–
3d	WPLP	SBC	IU	0.864	–	–	–	–
4d	WPLP*	Interviews	IU	0.880	–	–	–	–
5d	Lin et al. (29)	Aix-MARSEC	IU	0.910	–	–	–	–
6d	Roll et al. (32)	TAL	IU	0.758	–	–	–	–
7d	Roll et al. (32)	Aix-MARSEC	IU	0.889	–	–	–	–
8d	Roll et al. (32)	SBC	IU	0.638 (**0.870)	–	–	–	–
9d	Roll et al.* (32)	Interviews	IU	0.797	–	–	–	–
(*e*) *Zero-shot (out-of-domain) over training-set*								
No.	Training Set	Validation Set	Task	IU	Emphasis	Comma	Period	Question
1e	TAL	Interviews	IU,Emphasis,Prototype	0.652	0.689	0.541	0.275	0.533
2e	WPLP*	Interviews	IU	0.880	–	–	–	–
3e	Aix-MARSEC (53)	Interviews	IU,Prototype	0.834	–	0.930	0.583	–
4e	SBC (28)	Interviews	IU,Prototype	0.806	–	0.892	0.500	0.667

Unless otherwise noted, the training set and validation set are drawn from the same domain. (a) Performance of five model sizes of our tool, the WPLP (per-Word Prosodic Label Predictor) WHISPER variant; (b) Performance of the WPLP multitask disentanglement vs. single tasks; (c) Performance of three WPLP models that were trained and validated on their respective datasets; (d) WPLP performance vs. two available baselines. Note that for the results on TAL, Aix-MARSEC and SBC datasets were obtained by training and validation on the same dataset, whereas on Interviews, a validation-only set, we show the best obtained results, over all training sets and model sizes (marked by *); Row 8(d) presents the results of our implementation of ref. 32 and the results reported in ref. 32. (e) Zero-shot performance on the interviews validation set: the impact of three training sets.

^†^We use Aix-MARSEC boundary strength annotations to assess out model’s performance for prototype.

**Fig. 4.**
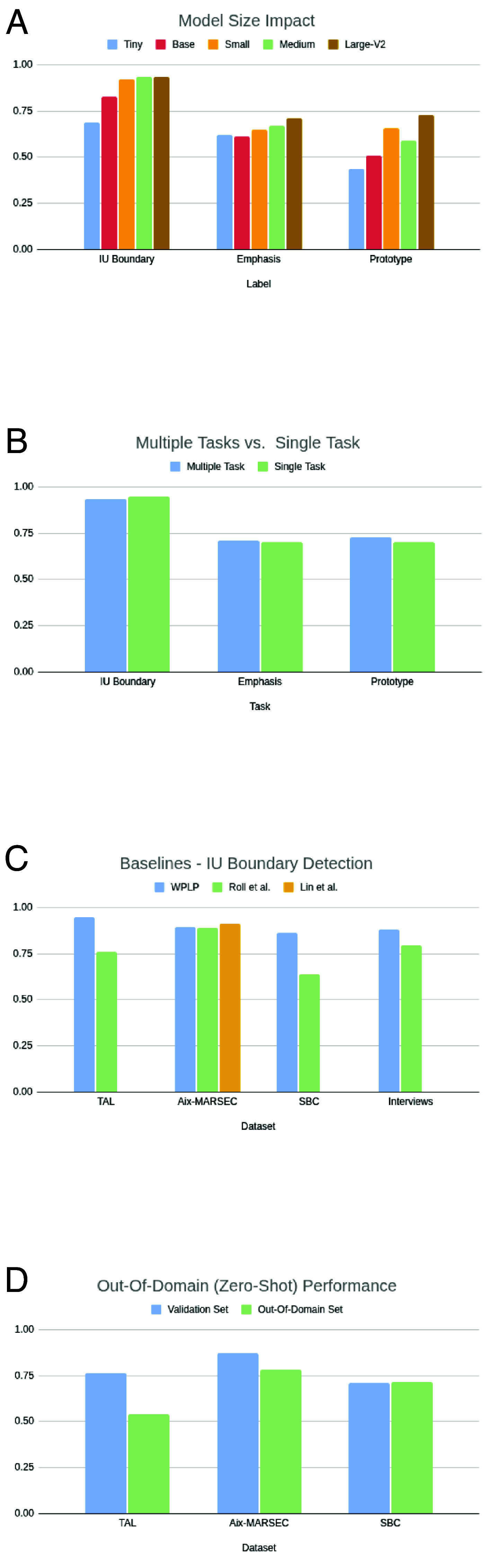
Results and comparisons: F1 scores for various detection tasks, models, and datasets. (*A*) Impact of the size of the pre-trained model on performance. F1 metric for IU boundary and emphasis detection, and macro-F1 for prototype detection, over the three alternatives. (*B*) Performance of the multi-task vs. the single-task frameworks. F1 metric for IU boundary and emphasis detection, and macro-F1 for prototype detection. (*C*) Performance of IU boundary detection, F1 metric: three baselines: WPLP, (29) and (32), against four datasets [(28, 53, 82) and the Interviews dataset (100)], and see also Sub-table (d) in Table 2. (*D*) Performance of WPLP on an out-of-domain (zero-shot) test set, F1 metric: a comparison of performance of three models, trained on three datasets (28, 53, 82). Labels are weighted equally.

For prototype recognition, the most prevalent prototype—continuation[,]—was best recognized when employing the WPLP WHISPER-Large V2, and when the model was trained for detecting IU prototypes only (Table 2, row 5a vs. row 4b). Counterintuitive, though, is the negligible (if any) difference between the performances on the single task vs. the multiple task (see Fig. 4*B*, which speaks for the robustness of the TAL-triple model.

As for model size, unsurprisingly and generally speaking, the larger the model, the better the performance (Table 2 rows 1a to 5a; Fig. 4*A*. That said, the improvement rate degrades rapidly. The Large V2 model performed significantly better on prototype detection, but across the majority of tasks, improvement between the small and the large models was not overly significant.

#### Other Methods/Baselines.

Results of our method vs. other methods are listed in Subtable (d) (see also Fig. 4*C*). We implemented the method in ref. 32 as a baseline for IU boundary-detection and ran it on our own datasets, as well as on the SBC. On the SBC validation set we obtained much lower results than the ones reported in ref. 32 (See row 8d, marked with **), which may be due to the exclusion of disfluencies in the preprocessing procedure of PSST (32). Despite the similarity in infrastructure and the fact that it labels less prosodic information than our method, the PSST performance is weaker in some cases—see Table 2, rows 1d vs. 6d and 4d vs. 9d, and see also rows 5d vs. 2d, showing the strength of ref. 29. On the Aix-MARSEC dataset, however, our implementation of ref. 32 obtained excellent results.

Overall, across datasets, our method outperforms (32), and in most cases by a considerable difference. The method described in ref. 29, though, obtained better results than ours on Aix-MARSEC (*Discussion*).

#### The Aix-MARSEC-trained model.

This model, trained for reference on a double recognition task (IU boundary and two prototypes), performed exceptionally well on prototype recognition (Table 2, row 2c).

### Out-of-Domain Performance.

Subtables (d) and (e) in Table 2 and Fig. 4*D* present out-of-domain (or zero-shot) performance. The tests were carried out on the Interviews dataset, which was excluded from the training material. Reporting such a direct experiment of domain shift is uncommon in the literature (cf. ref. 93 for a discussion of the effect of in- and out-of-domain data on pretraining and fine-tuning). Our results show some decrease in overall performance: macro-F1 of the Aix-MARSEC-trained model, compared to its results on its own validation set, degrades by 9% macro-F1 (from 87.2 to 78.2%; see rows 2c vs. 3e). The SBC-trained model obtains equivalent macro-F1 performance. We also see high variability across prosodic labels (see especially, 4e vs. 3c—high fluctuations for comma, period, and question). When shifting from in-domain to out-of-domain, we consider such a decrease to be a confirmation of a model’s resilience (see refs. 94–96).

Interestingly, we note a sharp decrease in performance for the TAL-trained model (rows 1c vs. 1e, 76.5% to 53.8% macro-F1). Two factors may be the reason for this gap: first, the annotation process of the TAL data, which relied more heavily on syntactic cues than that of the Interviews, SBC and Aix-MARSEC data. Second, the annotation of TAL induced significantly shorter chunks than other datasets, which, in turn, may have affected its performance.

Our results underscore the generality of our method also in regard to English dialects: although both TAL and Interviews are American English datasets, a model that was trained on British English (Aix-MARSEC) performs better on Interviews (mostly American English) than the one trained on American English (TAL).

### Impact of Prosodic Encodings.

The Compact encoding proved to be the most efficient representation with regard to execution time. That said, the feedback for this encoding during training was not ideal: since a single token stands for three labels, the model receives negative feedback unless all three labels are detected correctly. Conversely, the Bits encoding guaranteed adequate feedback to the model for each prosodic phenomenon, independently of the others. However, since the WHISPER architecture computes labels serially, it cannot consider a combination of feedbacks. In addition, the Bits encoding had a negative impact on execution time. This is due to the length of the sequence, which is doubled, compared to that of the Compact encoding.

### Results: Concluding Remarks.

Our results indicate that the WHISPER WPLP variant models separate well three different prosodic simultaneous messages. The system proved to be quite robust: in spite of its instability for some prosodic labels, the TAL-trained models generalize well over a large variety of speakers, for both spontaneous and read speech. As to out-of-domain performance, we consider the models that were trained on Aix-MARSEC and SBC to be satisfactory both for multitask/multilabel and for the two English dialects examined.

As the field of prosody disentanglement and function-based recognition is a recent one, it may be useful to cite interannotator agreement scores that are more current than the F1 metric: *SI Appendix*, Table S3 provides Cohen’s Kappa scores [henceforth CK (97)] for the TAL-trained V2 model, compared to those cited in ref. 18.

Well within the range on similar tasks, the TAL-trained V2 model achieves 0.893 CK on IU boundary recognition; 0.588 on emphasis detection; and 0.459 on prototype detection. For reference, in ref. 18, CK for IU boundary annotation is between 0.52 and 0.78; for pitch accent annotation, it is 0.71; and for the ToBI boundary size, CK is 0.47 to 0.68. Our method thus outperforms human annotators on the task of IU boundary detection (and see additional results in *SI Appendix*, Table S7); the TAL-trained triple model and single models perform on a par with, or superior to, human annotators on similar tasks (see also ref. 12; for a comparison of a ground-truth tagged text and automatically labeled one, see the *SI Appendix*, Fig. S3).

## Discussion

We have shown that co-occurring prosodic messages of different nonverbal dimensions may be disentangled and detected simultaneously. This is an encouraging validation of our multilayered approach to TIU prosodic patterning. The fact that the triple detection task yields similar results to the single task ones, and the modest amount of data required for triple recognition, constitute further corroboration of the role that TIUs play as useful domains for the detection of prosodic messages.

We have also shown the resilience of our models when dealing with out-of-domain data and with data of two different English dialects, American and British.

In addition, it seems that avoiding the conjoint task of transcription and prosody recognition is an advantage: despite the similarity in infrastructure and the fact that it labels less prosodic information, the performance of the PSST method (32), in some cases, is weaker. As may be expected, by overriding the transcription phase, prosody recognition improves.

In comparison, the authors of ref. 29 created a specifically tailored solution for the ToBI-annotated Aix-MARSEC dataset, with excellent results. Their feedback mechanism seems to be key to this success, an element that needs to be improved in our method. However, as opposed to ref. 29, our proposed solution is an easily implemented, almost off-the-shelf one, whose performance is robust on concurrent tasks.

Indeed, our aim here is less to achieve better prosody recognition, but, rather, to concentrate on the simultaneous detection of several prosodic functions. This problem is a fascinating one, and the model’s performance is a welcome by-product. Despite the difficulty in training for three different detection tasks at a time, and on a relatively small amount of diverse data, tagged by different annotators, the results are either on a par with, or superior to, that of average human annotation (cf. refs. 12 and 18, although these schemes are only partially function based). Thus, our WHISPER variant model can be considered an adept annotator for the prosodic phenomena learned.

In addition, we presented a method for multilabel, multiclass transfer learning that enriches the sequence of ASR transcription with prosodic labels. This “dynamic tokenizer”—a fine-tuning process that forces WHISPER into performing a new task using unfamiliar token combinations—seems to draw out information that already exists within the weights of the original model. As far as we are aware, this method of multiclass/multilabel transfer learning and a triple task has not been used for prosody analysis.

The present work is a step toward a larger-scale study and further disentanglement, a process that we have already begun for five potentially simultaneous sentiments.

There are many questions and challenges that we leave for future work. They can be divided into six main types:

1. In the domain of linguistic theory, improving the way to determine which prosodic messages are mutually exclusive, i.e., belong in the same category, and which are not. An example can be found in the nested pattern of the list within the apodosis in Fig. 1. Prosodic disentanglement can better define the various nonverbal dimensions, linguistic and paralinguistic, and establish systematic classification trees for TIUs.
2. Defining what constitutes a distinctive feature for prosodic patterning on the scale of IUs. An exploration of the model and its internal representations could be directed at determining, and eventually using, such features for a heuristic method of prosody classification (cf. ref. 98).
3. Describing the relationships of prosodic patterns to other linguistic components, and developing a tool for context formalization.
4. Developing a formal “language” or sign-system for the annotation of prosodic functions/meaning, to be affixed to text, in addition to the common written punctuation.
5. Extending the repertoire of reliably recognized prosodic patterns, including emotions and speaker attitudes, in addition to exploring prosodic universals vs. language- or community-specific phenomena.
6. Improving context learning for multiple prosodic tasks using a better multitask learning framework and exploring further architectures and encoding methods.

This attempt at the disentanglement of prosodic messages, based on IU analysis, has the potential of expanding the horizons of speech and language descriptions, and of supporting the long-standing efforts on context elucidation.

## Supplementary Material

Appendix 01 (PDF)

## Data Availability

Sample audio files have been deposited in This American Life (https://a7ce520753ab849816faaa3f4fc591b1.cdn.bubble.io/f1734958781238x469836983677240900/By%20throwing%20her%20napkin%20down_%20Bursting%20into%20tears_%20Running%20from%20the%20room_.wav) (99). Anonymized sample audio files have been deposited in Interviews dataset (https://paper-10.bubbleapps.io/version-test/paper_1_1_interviews) (100). Previously published data were used for this work (28, 53).

## Footnotes

^*^The lab-tagged TAL subset is available upon request and is subject to a material transfer agreement with the Weizmann Institute of Science.
