# Supplementary material for: Disentanglement of prosodic meaning: Toward a framework for the analysis of nonverbal information in speech
Source: Proc Natl Acad Sci U S A. 2025 Sep 12;122(37):e2500510122. doi: 10.1073/pnas.2500510122 (PMC12452892; doi:10.1073/pnas.2500510122)
Supplement: Supplementary file 1 — Appendix 01 (PDF) [file pnas.2500510122.sapp.pdf]

Supporting Information for

Disentanglement of Prosodic Meaning: Towards a Framework for the Analysis of Non-verbal Information in Speech

Tirza Biron, Moshe Barboy, Eran Ben-Artzy, Alona Golubchik, Yanir Marmor, Assaf Marron, Smadar Szekely, Yaron Winter, David Harel

This PDF file includes:

Supporting text
Tables S1 to S7
Figs. S1 to S5
Legends for Dataset S1 to S2
SI References

Other supporting materials for this manuscript include the following:

Datasets S1 to S2

Supporting Information Text

1. Prosodic analysis: details of the framework

In the interest of smooth reading, we recapitulate here the principles of our theoretical framework. Its categories are seen as applicable across datasets and speech communities, whereas sub-categories/individual labels are considered to be dataset specific. At its heart is a hierarchy of IU patterning.

Three premises direct the proposal:

(i) that an IU is a suitable unit for identifying intelligible, cohesive prosodic messages. It is a functional speech entity on a timescale that is similar to a phrase, and thus more readily interpretable than its parts (e.g., syllables);

(ii) that IUs convey communicative functions – semantically meaningful, sometimes grammaticalized, prosodic patterns ((1), (2), e.g., chapter 4);

(iii) that *"prosodic means [—] are often encoded by modifying existing forms that are already specified by other functions"* (3).

The "existing forms" in this context are 3-4 IU prototypes that exhibit meaningful prosodic variations, thus guiding the listener to infer the speaker's intentions. By *prototype* we mean an underlying pattern that is an attribute of every IU, and which is a pre-requisite for every additional prosodic information (cf., (4), see Figure 3 in the *main text*). The IU Prototype indicates the type of each IU: "continuation", "conclusion", or "request for response", denoted respectively by a comma, a period or a question mark. For this basic entity of an IU that is paired with its prototype, we propose the term "Typed-IU" (henceforth TIU). In our data we were able to identify 3-4 TIU templates.

The TIU is interpreted within a set of pre-established alterations, based on the listener's prior knowledge of the TIU template.

Each TIU can convey 3-8 (but no more) dimensions of variation that can then be classified into linguistic and paralinguistic categories. The former include "Discourse function", "Conversation action", and "Emphasis/information structure", and the latter sentiment-related ones: "Emotion" and "Attitude".

A. Linguistic functions.

A.1. Prosodic prototype: para-syntax/modality and TIU. Identical syntax and lexicon may carry different meanings: along with its fundamental role in dividing speech into intervals, prosody assigns every IU a prototype – a category that has been often termed para-syntax, or modality (5–8)). In our framework, the prototypes roughly correspond to boundary tones as defined in the SBC (9), for example, where they are marked up as follows: (1) flattish tone – comma; (2) falling tone – period; (3) rising tone - question mark; (4) and a fourth category - the truncated unit. The latter designates disfluencies in speech, or IUs that did not reach their 'natural' conclusion (cf. (10)).

Indeed, para-syntactic/prototype classes are often recognized by characteristic prosodic parameters that occur early and/or late in the unit (e.g., duration, intonation and intensity patterns). For our purposes, however, due to the multi-layered nature of prosodic messages, IU prototypes are tagged based on their *function* as experienced by the annotator, not their form, using the above mentioned commonly accepted punctuation marks. Our current sets of American English include two sub-types for the "request for response" [?] prototype: polar and WH questions. IUs that do not neatly conform to this scheme are relatively rare, $\leq$ 10%, and are none the less assigned one of the prototypes.

The following examples no. 1, 2, and 3 present the category of TIUs (examples no. 1 and 2 are cited from (8)):

[You want to go home] + [?] = You want to go home? [1]

[You want to go home] + [.] = You want to go home. [2]

[You want to go home] + [,] = You want to go home,
take a shower,
have a glass of wine... [3]

We contend that a vast majority (over 90%) of the examined data (9, 11) of some 630 hours of recorded speech may be classified into these primary categories.

A.2. Discourse Function and Conversation Action. This category is viewed as closely related to the prototype one. Clearly, the prototypes "continuation" [,], "conclusion" [.] or "request for response" [?] play a significant role in discourse organization and in conversation action. Yet, prosody provides additional information as to discourse structure and to a wide array of speech acts.

Each of the prototypes can be assigned a potential prosodic classification tree. In addition to the role that an utterance plays in social interaction, it determines the relationship of one TIU with other TIUs when forming larger discourse units. Consider example 4:

"you snooze,/ you lose" [4]

The if-clause (protasis) will usually employ the "continuation" [,] prototype, whereas the then-clause (apodosis) will use the "conclusion" [.] one. A "conclusion" [.] TIU will rarely appear at the beginning of a list, or as the first item of an apposition. It

will most often appear as the final item of a list, and/or when making a concluding stipulation. Other discourse functions are more flexible: parentheticals, for example, may take on any prototype.

This category is one of the most elaborate ones and perhaps the subtlest and hardest to tag (Table S4). In traditional grammar, it is positioned between syntax and rhetoric.

Conversation action vs. discourse function:

One might argue both for and against the distinction between the category of conversation action and that of discourse function. Indeed, for good measure, it would be best if one could neatly distinguish between the two. However, this is often not the case. The bottom label in Figure 1 in the *main text*, for example - "making a point" - describes the prosodic message of speaking with the intent of convincing, using a well-formed argument. The speaker clearly organized her discourse to culminate in a trio of crashing rhetorical questions. It can thus be classified as conversation action ("I aim to influence your opinion") and/or discourse organization/function ("hence, I use a reasoned contention").

There are clear-cut cases, such as example no. 4:

"you snooze,/ you lose" [4]

The bi-partite (=discourse organization) can also be labelled as e.g., a criticizing comment or a (gentle) scolding (=conversation action). However, once again, the distinction is not so obvious when considering another example, a list pattern (example no. 5):

"we had a drink, watched the sunset, chilled, [THEN ALL OF A SUDDEN—]" [5]

The list pattern (=discourse function) in this short narrative (=Discourse function? Conversation action?) prepares the ground (=Conversation action? Discourse function?) for a surprise narrative event. Thus, many of the terms that we apply when standardizing prosodic labels are work in progress.

Rhetorical questions are another convenient illustration of this blurred boundary.

Consider example no. 6:

Could you pass me the salt please? [6]

In polite society, the response to this question is not expected to be "yes" or "no", but the passing of the salt. It is a request for a physical gesture, rather than for information. In this case, the prototype "question" will be further labeled as a request in the conversation action column. Warnings, promises and performatives have their own prosodic patterns and are also included in this category. Another usage involves, again, a rhetorical question that is employed as an ambivalent element - a question that is not a question - to express unmet expectations:

You're not coming? [7]

Thus, as it may call for a change in the interlocutor's behavior, examples no. 5 and 7 may deserve a spot in the "conversation action" category. However, example no. 7 can also be labeled in the "discourse function" column under rhetorical question (cf. e.g., (12)).

The scope of prosodic discourse structures: Discourse constructions span from a sentence to a paragraph and further; from short appositions –

Arthur,/ the king of England,/ [8]

through longer structures such as a list of items:

Arthur,/ the queen of England,/ and Joe,/ (rode into the sunset). [9]

As already mentioned, a common example is bi-partite constructions, such as ["if"/"then"] and ["when"/"then"], for example,

Touch it,/ and you're dead. [10]

Longer combinations are, for example, a series of symmetrical statements that form the first part of a bi-partite, followed by its second part and resolution:

I don't want you to sit,/
I don't want you to stand,/
I don't want you to wiggle,// [11]
I just want you,/
to sit still.//

The items in example no. 11 make up a list that prepares the ground (i.e., first part of bi-partite) for the point made in the fourth and fifth units. Those, in terms of conversation action – and depending on their prosody – may be an instruction, a directive, or a warning (cf. (9): "I don't care if you're African-American,/ I don't care if you're Asian American,/ or whatever,// American minorities,/ do not vote,/ in great numbers.//". The audio excerpt can be found here.)

The longest prosodic constructions that we have encountered in spontaneous speech are argumentation passages and narratives: an introduction followed by details (e.g., a list of examples), then a point being made, and finally, a conclusion or a summary. Narratives present complex structures as well, for example: background, a concatenation of events that is intercepted by asides/parentheticals, followed by a conclusion. These larger structures would require a matryoshka-like analysis that would reflect their nestled nature.

It must be noted that the interpretation that we offer for prosodic discourse organization is, as of yet, incomplete: there are additional sub-categories and properties of TIUs, as well as inter-relations between sub-categories, that need defining and refining. Several examples, cited from our data sets, can be found in Table S4.

A.3. Information structure. An easily recognisable category that distinguishes more from less salient information.

You want to go *home*?! [12]

The emphasized word *home* is singled out as the most note-worthy information of the IU. Scholars distinguish various degrees of saliency (e.g., (13)).

B. Sentiment.

B.1. Attitude. Speaker intentional sentiment and/or attitude is perhaps the most sought-after result in language- and speech analysis. Mockery, sarcasm, or feigned anger at a child are conscious acts of communication that distinctly affect prosodic patterns. In our framework we acknowledge co-occurring messages within this category as well – e.g., a speaker may be assertive and empathetic at the same time (audio samples can be found here, taken from (14)).

Consider example no. 13:

I had so much fun last night. [13]

One cannot distinguish a sarcastic statement from a pleased one based on the text and without further context. However, upon hearing it, the speaker's intention becomes evident.

B.2. Emotion. Subconscious feelings are also naturally heard in speech prosody (audio samples can be found here, taken from (14)). When listening to a loved one, we can hear that they are upset or indifferent, tired or excited. Consider example no. 14, which may be produced in a variety of ways:

I think Max went home. [14]

The speaker could be angry ("How dare he go home now?!"), relieved ("Finally, he's gone!"), or disappointed ("What a shame… I so wanted to talk to him"), and so on. For emotion classification systems see (15, 16). Other emotions may be added to the list, for example, admiration, annoyance, or boredom, as well as their intensity (e.g., impatient, mildly annoyed, irritated, angry, very angry, enraged). For a suggestion of labels, see (17).

In summary, the annotation scheme that posits 5-8 categories and their sub-categories, is in an ongoing, fascinating process of standardization.

2. Producing Figure 3 in the Main Text

The plots in Figure 3 in the *Main Text* present the normalized median F0/pitch values in semitones for each time-normalized TIU of 4-7 word length. The median F0/pitch is represented by the dark shade, and the lighter shade represents the error bars.

A. Data Selection. The data used for Figure 3 consists of automatically obtained and annotated TIUs from the TAL dataset.

The selected length of TIUs was 4-7 words. This is due to two reasons: (i) shorter units may serve a different communicational purpose than 'standard' ones, and may not present a 'standard' pitch course; (ii) longer units often turn out to be segmentation errors (the average TAL IU contains a maximum of 7 words).

For Figures 3b-3e, each prototype group of automatically labeled TIUs was divided into two categories according to the position of emphasis - in the first half vs. the second half. TIUs in which emphasis occured in mid-unit were excluded.

Figures 3a and 3d-3e: TIUs of the question prototype were automatically divided into WH and polar questions (cf. (18)), based on their syntax. TIUs whose syntax was undetermined were excluded.

B. F0/Pitch Extraction and Normalization. F0/pitch was extracted using the Parselmouth Python library (19), at a sample rate of 100/sec. F0/pitch zero values were then removed, as they usually indicated voiceless consonants rather than non-speech chunks.

A median-based normalization of F0/pitch per speaker was calculated in semitones, to better represent the speaker's 'comfort zone' and to emulate the human prosodic experience.

C. Time Normalization. IUs were time-normalized 0-1 thus:

$$Time\_norm = \frac{timestamp - beginning\_time}{ending\_time - beginning\_time} \quad [15]$$

Then, each time-normalized IU was divided into 20 time-normalized slices.

D. Creating the Plots. Each time-normalized IU slice was assigned its respective normalized F0/pitch values, then represented by its first occurring F0/pitch value.

To produce all figures, the normalized F0/pitch values were merged with the timestamps of the words and their respective prosodic annotations, i.e., timestamps prior to time normalization.

3. Inter-annotator Agreement

As mentioned in the *main text*, to examine inter-annotator agreement for the three categories at hand, we used the Interviews dataset (20). The annotators independently transcribed and tagged the audio, marking unit boundaries (Yes/No), assigning a unit prototype (Comma, Period, or Question), and freely tagging emphasised words. Annotations were then manually compared, and agreement percentages were calculated for each category, speaker and turn (see Tables S1 and S2):

- **Unit boundaries:** ratio of agreed boundaries to the total number of annotated boundaries;
- **Prototypes:** ratio of agreed prototypes to the number of agreed unit boundaries, similarly to the score calculation for automatic prototypes recognition (see the “Evaluation” sub-section in the *main text*);
- **Emphases:** ratio of agreed emphases to total annotated emphases. Unlike the other two categories – which have strict annotation rules (yes/no for unit boundaries and a closed set of three for prototypes) – emphasis tagging was more flexible, taking into account its varying degrees and functions. One annotator distinguished between "weak" and "strong" emphases while the other distinguished between "strong" and "very strong" ones. For the purposes of this paper, agreement was noted when both used "strong," or when one used "strong" and the other used either the "weak" or "very strong" annotation. Cases in which one annotator tagged a word as bearing “very strong” or “strong” and the other did not find an emphasis at all, were considered as disagreements. If one annotator marked a word as "weak" and the other left it untagged, the word was noted as un-emphasized and excluded from the count.

The annotators jointly reviewed snippets with less than 70% agreement in at least one of the parameters, correcting the text – mainly disfluencies - and refining the tags as needed - carefully avoiding bias. Final inter-annotator agreement reached Cohen’s Kappa of 0.866 for unit boundaries, 0.728 for prototypes, and 0.833 for emphasis.

4. Cohen’s Kappa (CK) scores for automatic vs. manual annotations

Table S3 presents CK scores for several automatic and manual annotations and datasets. In rows 4-7 are CK values for the Interviews dataset, which is the only set to be annotated by two annotators. Note that these data were used for zero-shot testing only, not as a regular test set; hence, the discernible - and expected - degradation in performance.

Table S1. Interviews dataset, Inter-annotator agreement per turn

Turn no.	Turn name	Word count	Boundary				Prototype				Focus			
			Agreed	All	%	K	Agreed	All	%	K	Agreed	All	%	K
1	Arnold 1	66	17	19	89.5	0.97	17	17	100.0	1.00	18	21	85.7	0.95
2	Arnold 2	99	21	25	84.0	0.96	21	21	100.0	1.00	20	25	80.0	0.95
3	Arnold 3	58	13	17	76.5	0.93	12	13	92.3	0.92	19	21	90.5	0.97
4	Arnold 4	48	12	13	92.3	0.98	12	12	100.0	1.00	18	19	94.7	0.98
5	Koval 1	79	17	23	73.9	0.92	16	17	94.1	0.94	22	23	95.7	0.99
6	Obama 1	46	12	16	75.0	0.91	12	12	100.0	1.00	13	14	92.9	0.98
7	Obama 2	214	59	66	89.4	0.97	58	59	98.3	0.98	57	61	93.4	0.98
8	O'Brian 1	63	13	19	68.4	0.90	13	13	100.0	1.00	16	17	94.1	0.98
9	O'Brian 2	35	7	10	70.0	0.91	6	7	85.7	0.86	6	9	66.7	0.91
10	O'Brian 3	39	7	10	70.0	0.92	7	7	100.0	1.00	8	10	80.0	0.95
11	O'Brian 4	41	11	12	91.7	0.98	11	11	100.0	1.00	9	13	69.2	0.90
12	O'Brian 5	35	8	10	80.0	0.94	8	8	100.0	1.00	9	9	100.0	1.00
13	O'Brian 6	37	5	6	83.3	0.97	3	5	60.0	0.60	8	9	88.9	0.97
14	O'Brian 7	79	25	28	89.3	0.96	25	25	100.0	1.00	21	25	84.0	0.95
15	Oprah 1	91	21	22	95.5	0.99	20	21	95.2	0.95	18	39	46.2	0.77
16	Oprah 2	32	8	8	100.0	1.00	8	8	100.0	1.00	10	10	100.0	1.00
17	Oprah 3	66	16	18	88.9	0.97	16	16	100.0	1.00	20	22	90.9	0.97
18	Oprah 4	38	8	8	100.0	1.00	8	8	100.0	1.00	11	11	100.0	1.00
19	Oprah 5	84	14	20	70.0	0.93	14	14	100.0	1.00	17	27	63.0	0.88
20	Smith 1	23	7	8	87.5	0.96	7	7	100.0	1.00	6	6	100.0	1.00
21	Smith 2	41	16	17	94.1	0.98	16	16	100.0	1.00	12	14	85.7	0.95
22	Smith 3	23	10	13	76.9	0.87	10	10	100.0	1.00	9	11	81.8	0.91
23	Smith 4	61	16	20	80.0	0.93	15	16	93.8	0.94	15	19	78.9	0.93
24	Smith 5	81	17	20	85.0	0.96	17	17	100.0	1.00	18	23	78.3	0.94
25	Smith 6	66	22	26	84.6	0.94	21	22	95.5	0.95	16	30	53.3	0.79
26	Watson 1	65	12	14	85.7	0.97	11	12	91.7	0.92	11	19	57.9	0.88
27	Watson 2	134	20	28	71.4	0.94	19	20	95.0	0.95	26	30	86.7	0.97
28	Watson 3	66	13	15	86.7	0.97	11	13	84.6	0.85	12	23	52.2	0.83
29	Watson 4	50	12	14	85.7	0.96	11	12	91.7	0.92	12	18	66.7	0.88
30	Watson 5	100	20	23	87.0	0.97	18	20	90.0	0.90	22	23	95.7	0.99
	**Overall**	**1960**	**459**	**548**	**83.8**	**0.95**	**443**	**459**	**96.5**	**0.97**	**479**	**601**	**79.7**	**0.94**

Table S2. Interviews dataset (20), Inter-annotator agreement per speaker

Speaker	No. of files	Word count	Boundary				Prototype				Focus			
			Agreed	All	%	K	Agreed	All	%	K	Agreed	All	%	K
Arnold	4	271	63	74	85.1	0.96	62	63	98.4	0.98	75	86	87.2	0.96
Koval	1	79	17	23	73.9	0.92	16	17	94.1	0.94	22	23	95.7	0.99
Obama	2	260	71	82	86.6	0.96	70	71	98.6	0.99	70	75	93.3	0.98
O'Brian	7	329	76	95	80.0	0.94	73	76	96.1	0.96	77	92	83.7	0.95
Oprah	5	311	67	76	88.2	0.97	66	67	98.5	0.99	76	109	69.7	0.89
Smith	6	295	88	104	84.6	0.95	86	88	97.7	0.98	76	103	73.8	0.91
Watson	5	415	77	94	81.9	0.96	70	77	90.9	0.91	83	113	73.5	0.93
**Overall**	**30**	**1960**	**459**	**548**	**83.8**	**0.95**	**443**	**459**	**96.5**	**0.97**	**479**	**601**	**79.7**	**0.94**

Table S3. Cohen's Kappa scores for several annotation tasks, automatic 1-6 and manual 7-8. For reference, line 8 cites the scores provided in (21), although the tasks are not identical (*prosodic boundary annotation; ** pitch accent (not emphasis) annotation ; * boundary size). Note that the Interviews data were used for zero-shot testing only, hence the lower performance.**

	Train Set	Test Set	IU	Emphasis	Prototype
1	TAL	TAL	0.893	0.588	0.459
2	Marsec	Marsec	0.844	-	0.731
3	SBC	SBC	0.785	-	0.497
4	TAL	Interviews	0.571	0.570	0.159
5	Marsec	Interviews	0.776	-	0.512
6	SBC	Interviews	0.739	-	0.476
7	Annotator I-Annotator II	Interviews	0.866	0.833	0.728
8	(21)		0.52-0.78*	0.71**	0.47-0.68***

Table S4. ***Examples for discourse function labels by prosodic prototype.*** **Audio samples.**

**Categories of non-verbal information**	**Sub-categories of non-verbal information**	**Continuation prototype [,]**	**Conclusion prototype [.]**	**Question prototype [?]**
Apposition		These enzymes,*	Of making pharmaceuticals.*	What's the strangest product?*
Bi-partites	Conditional	You have no guide,† (Protasis)	When you're a young person.† (Protasis)	Why shouldn't you steal? Why shouldn't you shoot? Why shouldn't you go to prison?† (Apodosis)
Conclusion		You have no guide,† (Beginning of conclusion)	When you're a young person.† (Continuation of conclusion)	Why shouldn't you steal? Why shouldn't you shoot? Why shouldn't you go to prison?† (Continuation/ending of conclusion)
Conversation action	Announcement	It's This American Life,‡	That's what I want to engineer.*	
	Confirmation		V	Right?*
	Explanation	The enzymes, are the catalysts,*	That do that magical transformation.*	
	Request	Can you explain why,*	V	anybody would want to do that?*
Lists (open/closed)	Closed	Shaky, out of focus, bad color,‡ (Non-final list item)	Over-blown color actually.‡ (Final list item)	By throwing her napkin down? Bursting into tears? Running from the room?‡ (Final/non-final list item)
Narration	Background	So a new girl transfers into Rebeca's high school,‡	An all-girl school.‡	V
	Main subject	That they propagated to,*	These methods are sufficiently simple.*	V
	Title	Here's what I did for fun,‡	Ghetto hoochie mama.‡	V
	About to make a point	Now,†	V	V
	Making a point	You have no guide,†	When you're a young person.†	Why shouldn't you steal? Why shouldn't you shoot? Why shouldn't you go to prison?†
	Point made		That's what I want to engineer.*	V
Parenthetical		Into living organisms,*	Although sometimes I get too competitive.*	Or not really?*
Rhetorical questions				Why shouldn't you steal? Why shouldn't you shoot? Why shouldn't you go to prison?†

* Interviews dataset (20).
† Santa Barbara Corpus (9).
‡ This American Life dataset (11).

Table S5. ***Examples for information structure (various emphases).*** **Audio samples, taken from (14).**

**Categories of emphasis**	**Continuation prototype** [,]	**Conclusion prototype** [.]	**Question prototype** [?]
Contrastive	But that's not how YOU perceive it,	THAT'S what I want to engineer.	Why SHOULDN'T you steal?
Strong/main	With people in PRISON,	Into EDUCATION.	Meaning she's from public SCHOOL?
Secondary	With PEOPLE in prison,	When you're a YOUNG person.	MEANING she's from PUBLIC shool?
De-emphasis	WITH people IN prison,	INTO education.	Meaning SHE'S FROM public school?

Table S6. ***Examples for sentiment (ordered from highest to lowest agreement).*** **Audio samples, taken from (14).**

**Text**	**Tagger1**	**Tagger2**	**Tagger3**	**Tagger4**
I was taking the gun	Distress	Distress	Distress	Distress, fear
We're chasing some of them right now	Empathy	Empathy	Neutral	Empathy
You need to tell me what area you were in	Confident, distress	Confident	Invalid	Confident
We've just been robbed	Distress	Distress, fear	Distress	Fear
Stay with me	Distress	Confident	Distress	Neutral
And my husband was finally able to get to his gun and he shot him	Fear	Sadness	Distress	Distress
He was the experienced flyer	Confident, distress	Fear	Distress	Confused
Okay, well, some else is talking to the deputies, how did they get here?	Confused	Confident	Neutral	Neutral

Table S7. Comparison of performance of two label encodings: Compact and Bits. F1 scores for various classification tasks of the fine-tuned WHISPER (22) models, and (23).

Test set	Train set	Model size	Representation	Task	IU	Emphasis	Comma	Period	Question
TAL	TAL	Large	Compact	IU, Emphasis, Prototype	0.935	0.710	0.769	0.720	0.688
TAL	TAL	Large	Bits	IU, Emphasis, Prototype	0.944	0.675	0.779	0.695	0.603
Marsec	Marsec	Large	Compact	IU, Prototype	0.886	-	0.930	0.799	-
Marsec	Marsec	Large	Bits	IU, Prototype	0.875	-	0.924	0.774	-
Aix-MARSEC	Aix-MARSEC	(23)	-	IU	0.910	-	-	-	-
Interviews	TAL	Large	Compact	IU, Emphasis, Prototype	0.646	0.573	0.596	0.338	0.571
Interviews	TAL	Large	Bits	IU, Emphasis, Prototype	0.701	0.573	0.691	0.423	0.636
Interviews	Marsec	Large	Compact	IU, Prototype	0.813	-	0.930	0.667	-
Interviews	Marsec	Large	Bits	IU, Prototype	0.820	-	0.931	0.667	-

[**bold:** emphasis]; [parenthetical]; [sound/recording issue]

[apposition]; [apodosis]; [title/opening statement]; [additional example]; [making unexpected Point]

Audio name: Arnold 2

these **methods** are **sufficiently** simple.// that they **propagated** to,/ **laboratories** all over the **world**.// for example,/ **Merck**,/ makes,/ some of their biggest **drugs**,/ using **enzymes**,/ that **replace** toxic metals,/ that they **used** to use in chemical processes,/ **really** reduced the **waste footprint**,/ of making pharmaceuticals.// the **enzymes** you find in your **laundry** detergent,/ that take **stains** off **clothes**,/ -- **nature** never made an enzyme,/ that likes to work in **laundry** detergents,/ but people **bred** them,/ using my **methods**,/ a--- to work very **well**.// enzymes help you,/ use much less **energy**,/ so,/ you,/ you don't have to use high **temperatures**.//

Fig. S1. An example of a detailed annotation for the linguistic categories of TIUs and discourse functions. See the audio file "arnold_02" here, taken from the Interviews dataset.

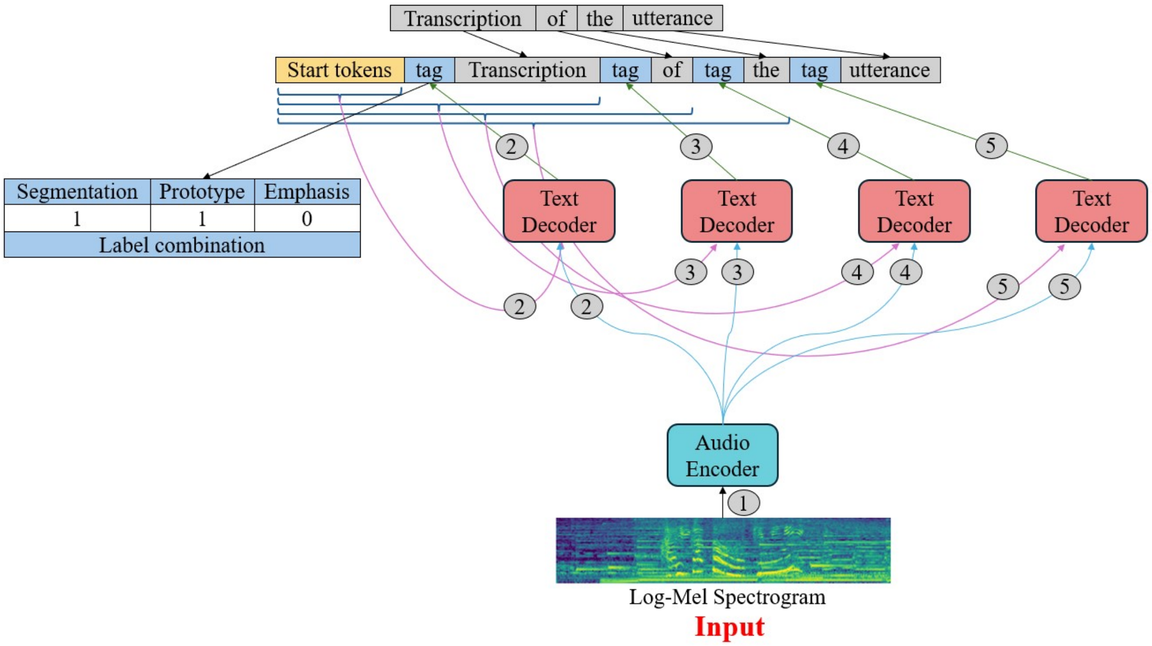

Fig. S2. WHISPER (22) training and inference schemes. To fine-tuned WHISPER, input includes speech audio, its corresponding text and prosodic tags; output predicts label combinations for each word of the input text. The numbers indicate the order of steps. As with any transformer, each decoder-step generates the next token, which is required for the subsequent step.

Crouch and **lift** at the same time,
Weight over the **balls** of your feet,
Now look,
I never knew my feet had **balls**.
I've **heard** the expression,
but I have never **contacted** them.
Basically,
I felt like a bad **geometry** class,
I felt all **fragmented**.

(a)

Crouch and lift at the same time,
Weight over the **balls** of your feet,
Now look,
I never knew my feet had **balls**.
I've **heard** the expression,
but I have **never** contacted them.
Basically,
I felt like a bad **geometry** class.
I felt all **fragmented**.

(b)

Fig. S3. Ground truth and output of the re-trained model. (a): An example of manually tagged audio and text; (b): The same example, model-labeled output (audio).

```
Require: words_table that includes the next columns: audio_file ← the correspondent audio signal
    start_time ← of the word w.r.t. the audio signal
    end_time ← of the word w.r.t. the audio signal
    segment_id ← induced by the IU boundary flag
Require: assumption that the table is sorted by
    < audio_file, start_time >
Require: init_pivot = pivot initialization function
    pending_chunk_start_row = current row index (0 at start)
    pending_chunk_start_time = start_time of the current row
Require: max_words_number = per the whisper limitation to 448 tokens
Require: max_valid_gap = max time gap between adjacent segments in a valid chunk
Ensure: start_chunk: indicates whether a word starts a new chunk
for row in words_table do
    if audio_file[row] != audio_file[row − 1] then
        init_pivot
        start_chunk = True
    else if segment_id[row] = segment_id[row − 1] then
        start_chunk = False
    else if accumulated_duration ≥ 30 seconds then
        init_pivot
        start_chunk = True
    else if accumulated_words _number >
            max_words_number then
        init_pivot
        start_chunk = True
    else if start_time[row] − end_time[row − 1] >
            max_valid_gap then
        init_pivot
        start_chunk = True
    else
        start_chunk = False
    end if
end for
```

Fig. S4. Speech-chunk compilation for training and testing

Require: Model: the re-trained model (based on WHISPER), which consists of an audio encoder and a text decoder.
Require: Tokenizer: converts text into the model's known tokens.
Require: Audio_spectrogram: audio in the format suited for the model's input (of the currently handled turn).
Require: Word_list: the words in the transcription, sorted by order of utterance.
Ensure: Label_list: the tags corresponding to the word list and aligned with them.

```
label_list ← empty list
token_list ← model's starting tokens
audio_features ← model.audio_encoder
        (audio_spectrogram)
for word in word_list do
    label_logits ← model.text_decoder(
            token_list, audio_features)
    next_label ← label with highest probability in
            label_logits
    append next_label to label_list
    append next_label to token_list
    append tokenizer.encode(word) to token_list
end for
return label_list
```

Fig. S5. Pseudo code of the prediction/inference procedure. This method enables prosodic labels to be drawn separately from the text. Note that *next_label* holds the predicted label of a multiclass-multilabel combination.

SI Dataset S1 (TAL)

A sample of the TAL dataset can be found here.

SI Dataset S2 (Interviews)

A sample of the Interviews dataset can be found here.

References

1. C Portes, C Beyssade, A Michelas, JM Marandin, M Champagne-Lavau, The dialogical dimension of intonational meaning: Evidence from french. *J. Pragmat.* **74**, 15–29 (2014).
2. E Couper-Kuhlen, M Selting, *Interactional linguistics: Studying language in social interaction.* (Cambridge University Press, Cambridge), (2017).
3. Y Xu, S Prom-on, F Liu, The penta model: Concepts, use, and implications in *Prosodic Theory and Practice.* (The MIT Press), (2022).
4. K Hirose, H Fujisaki, M Yamaguchi, Synthesis by rule of voice fundamental frequency contours of spoken japanese from linguistic information in *ICASSP'84. IEEE International Conference on Acoustics, Speech, and Signal Processing.* (IEEE), Vol. 9, pp. 597–600 (1984).
5. JW Du Bois, S Schuetze-Coburn, S Cumming, D Paolino, Outline of discourse transcription in *Talking Data.* (Psychology Press, New York), pp. 45–89 (2014).
6. W Chafe, *Discourse, consciousness, and time: The flow and displacement of conscious experience in speaking and writing.* (University of Chicago Press, Chicago), (1994).
7. J Mukherjee, *Form and function of parasyntactic presentation structures: A corpus-based study of talk units in spoken English.* (Brill, Amsterdam - Atlanta) Vol. 35, (2021).
8. SF Gardner, Ph.D. thesis (The University of St Andrews) (1985).
9. JW Du Bois, WL Chafe, C Meyer, SA Thompson, N Martey, Santa barbara corpus of spoken american english. *CD-ROM. Philadelphia: Linguist. Data Consortium* (2000).
10. BS Reed, Units of interaction:"intonation phrases" or "turn constructional phrases". *Actes/Proceedings from IDP (Interface Discours & Prosodie)* pp. 351–363 (2009).
11. I Glass, This american life (Chicago Public Media) (1995-present) [Online]. Available: https://www.thisamericanlife.org/archive.
12. K Wei, et al., A neural prosody encoder for end-to-end dialogue act classification in *ICASSP 2022-2022 IEEE International Conference on Acoustics, Speech and Signal Processing (ICASSP).* (IEEE), pp. 7047–7051 (2022).
13. WH Wells, An experimental approach to the interpretation of focus in spoken english in *Intonation in discourse.* (Routledge), pp. 53–76 (2018).
14. L Teitelbaum, G Allen, 911 recordings (Kaggle) (2023) [Online]. Available: https://www.kaggle.com/datasets/louisteitelbaum/911-recordings/data.
15. P Ekman, Are there basic emotions? *Psychol. Rev.* **99**, 550–553 (1992).
16. Humaine emotion annotation and representation language (http://emotionresearch.net/projects/humaine/earl) (2006) Accessed: 2024-12-18.
17. S Cenceschi, L Sbattella, R Tedesco, Calliope: A multi-dimensional model for the prosodic characterization of information units. *Estudios de fonética experimental* **30**, 227–245 (2021).
18. H Arndt, RW Janney, *InterGrammar: Toward an integrative model of verbal, prosodic and kinesic choices in speech.* (Walter de Gruyter) Vol. 2, (2011).
19. Y Jadoul, B Thompson, B De Boer, Introducing parselmouth: A python interface to praat. *J. Phonetics* **71**, 1–15 (2018).
20. BMGAMYSSHD Biron, T., The interviews dataset for prosody analysis (2025-present) [Online]. Available: https://theprosody.com/prosody_paper_3_1_interviews_dataset_references.
21. M Breen, LC Dilley, J Kraemer, E Gibson, Inter-transcriber reliability for two systems of prosodic annotation: Tobi (tones and break indices) and rap (rhythm and pitch). *Corpus linguistics linguistic theory* **8**, 277–312 (2012).
22. A Radford, et al., Robust speech recognition via large-scale weak supervision in *International Conference on Machine Learning.* (PMLR), pp. 28492–28518 (2023).
23. B Lin, L Wang, X Feng, J Zhang, Joint detection of sentence stress and phrase boundary for prosody. in *INTERSPEECH.* pp. 4392–4396 (2020).
